# The Guanine Nucleotide Exchange Factor Kalirin-7 Is a Novel Synphilin-1 Interacting Protein and Modifies Synphilin-1 Aggregate Transport and Formation

**DOI:** 10.1371/journal.pone.0051999

**Published:** 2012-12-20

**Authors:** Yu-Chun Tsai, Olaf Riess, Anne S. Soehn, Huu Phuc Nguyen

**Affiliations:** Department of Medical Genetics, University of Tuebingen, Tuebingen, Germany; Hungarian Academy of Sciences, Hungary

## Abstract

Synphilin-1 has been identified as an interaction partner of α-synuclein, a key protein in the pathogenesis of Parkinson disease (PD). To further explore novel binding partners of synphilin-1, a yeast two hybrid screening was performed and kalirin-7 was identified as a novel interactor. We then investigated the effect of kalirin-7 on synphilin-1 aggregate formation. Coexpression of kalirin-7 and synphilin-1 caused a dramatic relocation of synphilin-1 cytoplasmic small inclusions to a single prominent, perinuclear inclusion. These perinuclear inclusions were characterized as being aggresomes according to their colocalization with microtubule organization center markers, and their formation was microtubule-dependent. Furthermore, kalirin-7 increased the susceptibility of synphilin-1 inclusions to be degraded as demonstrated by live cell imaging and quantification of aggregates. However, the kalirin-7-mediated synphilin-1 aggresome response was not dependent on the GEF activity of kalirin-7 since various dominant negative small GTPases could not inhibit the formation of aggresomes. Interestingly, the aggresome response was blocked by HDAC6 catalytic mutants and the HDAC inhibitor trichostatin A (TSA). Moreover, kalirin-7 decreased the level of acetylated α-tubulin in response to TSA, which suggests an effect of kalirin-7 on HDAC6-mediated protein transportation and aggresome formation. In summary, this is the first report demonstrating that kalirin-7 leads to the recruitment of synphilin-1 into aggresomes in a HDAC6-dependent manner and also links kalirin-7 to microtubule dynamics.

## Introduction

Parkinson disease (PD) is the second most common neurodegenerative disease characterized by the degeneration of dopaminergic neurons of the substantia nigra and the accumulation of cytoplasmic inclusions known as Lewy bodies (LB) [1]. Mutations found in genes encoding α-synuclein [2], parkin [3], PTEN-induced putative kinase 1 (PINK1) [4], DJ-1 [5], Leucine-rich repeat kinase 2 (LRRK2) [6], and VSP35 [7], [8] are known to play roles in rare familial forms of PD, indicating that protein misfolding and aggregation, defects in the ubiquitin-proteasome system (UPS), mitochondrial dysfunction and cellular signaling are among other mechanisms involved in the pathogenesis of PD.

Synphilin-1, a protein of 919 amino acids, was identified as the first interactor of α-synuclein [9]. It contains several domains propagating protein-protein interactions, including ankyrin-like repeats and a coiled-coil domain, facilitating a role of synphilin-1 as a potential adapter molecule that could link α-synuclein to other intracellular proteins, which could be involved in vesicle transport, cytoskeletal functions, mitochondrial dysfunction and other pathogenetic mechanisms. Apart from interacting with α-synuclein, synphilin-1 also interacts with other proteins involved in the pathogenesis of PD, linking synphilin-1 to the ubiquitin-proteasome system and aggregation pathways. Specifically, synphilin-1 interacts with and is ubiquitinated by different E3 ligases, such as dorfin, SIAH-1 and SIAH-2 [10]–[12] marking synphilin-1 for the degradation via the UPS. Phosphorylation by the synphilin-1 interactor GSK3β is thereby a regulator of ubiquitination that leads to a decrease of synphilin-1 ubiquitination and inclusion formation [13]. The interaction with the proteasome subunit S6 ATPase/Tbp7 [14] and the negative regulator of ubiquitin-like proteins NUB1 [15] provides another link to proteasomal degradation. Parkin, which is most frequently mutated in early onset autosomal recessive forms of PD, is another interacting E3 ligase [16], however, it ubiquitinates synphilin-1 via K63-linked ubiquitin chains, thereby increasing the aggregation rate of the synphilin-1 protein [17].

Recently, we identified a novel R621C substitution in the *synphilin-1* gene in two apparently unrelated sporadic German PD patients that represents a potential susceptibility factor for PD leading to increased susceptibility to cellular stress *in vitro* [18]. To further explore the role of synphilin-1 in the pathogenesis of PD, we aimed to discover additional interaction partners using a yeast two hybrid screen identifying kalirin-7 as a novel interactor.

Kalirins are brain-specific guanine nucleotide exchange factors (GEFs) for Rho-like small GTPases, which were first identified as proteins interacting with peptidylglycine α-amidating monooxygenase [19]. Successively, an interaction with huntingtin-associated protein 1 (HAP1) was discovered [20], linking kalirins to Huntington disease (HD). Kalirins are encoded by the *kalirin (KALRN)* gene on chromosome 3q21.2 that generates several isoforms by alternative splicing and the usage of multiple promoters [21]. Rat kalirin-7, corresponding to isoform 2 of the human *kalirin* transcripts, is the major splice variant in the adult cortex. It is enriched in the postsynaptic density (PSD) of excitatory synapses [22], [23]. While it is undetectable at birth, the expression of kalirin-7 increases during synaptogenesis [21], [24]. Kalirin-7 contains a SEC14 domain, spectrin-like domains, a single RhoGEF domain, a pleckstrin homology domain (PH) and terminates with a postsynaptic density-95 (PSD-95)/Discs large/zona occludens-1 (PDZ) binding motif [19] which binds to PSD-95 [22]. With multiple domains and an abundance of interactions with other proteins, kalirin-7 could integrate various signaling inputs and modulate dendritic spine maturation, plasticity and dynamics. *In vitro* studies demonstrate that kalirin-7 acts downstream of surface adhesion molecules, such as ephrin receptors, NMDA receptors, N-cadherins and Trk receptors and is involved in the recruitment of AMPA receptors into spines [25]–[28]. The major signaling output of activated kalirin-7 is the enhancement of Rac1 activation which leads to the activation of p21 activated kinase (Pak) and a subsequent Pak-dependent actin cytoskeleton rearrangement [27], [29]. Kalirin-7 knockout mice show decreased anxiety-like behavior and a deficiency in hippocampal long-term potentiation [30] while total kalirin knockout mice also display some phenotypes relevant to psychiatric disorders [31]. Recently, several genome-wide association studies revealed genetic associations of the *kalirin* gene with coronary artery disease [32], ischemic stroke [33] and schizophrenia [34]. Furthermore, kalirin-7 is supposed to be involved in Alzheimer disease and schizophrenia due to an interaction with nitric oxide synthase (iNOS) [35], [36] and disrupted in schizophrenia 1 (DISC1) [34], respectively.

A functional overlap of kalirin-7 and synphilin-1 and thereby a first starting point to study a potential functional relevance of the newly discovered interaction lies in the formation of aggresomes. It is well established that synphilin-1 is able to promote inclusion formation, both in association with α-synuclein [9], [37] and alone [38]. Thereby, synphilin-1 forms multiple small highly mobile aggregates that are rapidly translocated into aggresomes upon proteasome inhibition which is dependent on its aggresome-targeting signal [39]. Also K63-linked polyubiquitination that is known to target proteins into the aggresome-autophagy pathway [40], [41] has been shown to enhance synphilin-1 inclusion formation [17].

Aggresomes are organelles that serve as storage compartments for misfolded proteins when molecular chaperones and the ubiquitin-proteasome system (UPS) become overwhelmed with damaged or misfolded proteins, but they can also be actively involved in the refolding or degradation of abnormal proteins. Aggresome formation is dependent on retrograde dynein-dependent trafficking along microtubules. However, the basic molecular mechanisms involved are poorly understood. Evidences indicate that aggresomes do not only associate with the dynein motor complex but also with histone deacetylase 6 (HDAC6) [40], ataxin-3 [42], and ubiquilin-1 [43] for recognition and transportation of cargo proteins and with chaperones, ubiquitination enzymes and proteasomes to facilitate the clearance of aggregated proteins [44]. Dynein-dependent transportation along microtubules can be regulated by Rho-GTPases [45] and therefore we investigated if kalirin-7 being a neuronal GEF for Rho-GTPases is involved in aggresome formation or turnover.

Indeed, our findings reveal a novel role for kalirin-7 in aggresome dynamics, namely in the HDAC6 dependent recruitment of small cytoplasmic synphilin-1 inclusions into aggresomes and thereby potentially link the protein for the first time to the pathogenesis of PD.

## Materials and Methods

### Yeast Two Hybrid Assays and Yeast Culture

The synphilin-1 bait construct was generated as a fusion construct encoding aa 177–348 and 557–920 in the pLEXA-DIR vector (Dualsystems Biotech AG, Switzerland). Thereby, the central ankyrin-like repeats, the coiled-coil domain, and the ATP/GTP-binding domain were excluded in order to prevent unspecific interactions. To avoid self-activation, the first 176 aa of synphilin-1 were truncated due to their acidic nature. Self-activation was tested by co-transformation of bait and control constructs. The bait construct was co-transformed with a human brain complementary DNA (cDNA) library into yeast strain L40 (MATa his3_200 trp1-901 leu2-3,112 ade2 LYS2::(lexAop)4-HIS3 URA3::(lex-Aop)8-lacZ GAL4) using standard procedures [46]. Positive transformants were tested by filter assays to validate β-galactosidase activity. Plasmids of positive clones were isolated and retransformed into L40 with the bait plasmid and a control bait encoding a LexA-lamin C fusion protein. Plasmids that showed β-galactosidase activity upon co-transformation with the bait plasmid but not the control bait were considered as bait-dependent positive interactors. Positive interactors were identified by sequencing.

### Reagents and Antibodies

Nocodazole, colchicine and trichostatin A (TSA) were purchased from Sigma-Aldrich. Sodium butyrate (NaBu) was from Enzo Life Sciences. Commercial antibodies used for western blotting (WB) analysis or immunofluorescence (IF) include rabbit anti-FLAG (WB, 1∶500; IF, 1∶200; Sigma), mouse anti-FLAG (IF, 1∶400; Sigma), mouse anti-V5 (WB, 1∶500; Sigma), rabbit anti-HA (WB, 1∶500; Sigma), rabbit anti-synphilin-1 (WB, 1∶1000; S5946, Sigma), goat anti-KALRN (WB, 1∶500; ab52012, Abcam), mouse anti-γ-tubulin (IF, 1∶100; clone GTU-88, Sigma), mouse anti-acetyl-tubulin (WB, 1∶4000; IF, 1∶200; clone 6-11-B1, Sigma), mouse anti-α-tubulin (WB, 1∶5000; clone B-5-1-2, Sigma), rabbit anti-ubiquitin (IF, 1∶300; Santa Cruz), Hsp27 (IF, 1∶200; C-20, Santa Cruz), and mouse anti-vimentin (IF, 1∶300; clone RV202, Abcam); all secondary antibodies were purchased from Amersham Biosciences.

### Plasmid Constructs

Human kalirin-7 [NM_003947.4] and synphilin-1 [NM_005460.2] cDNA were gifts from Richard E. Mains (Department of Neuroscience, University of Connecticut Health Center, Farmington, USA) and Frank P. Marx (Hertie Institute for Clinical Brain Research, Tuebingen, Germany), respectively. Both full length cDNAs and deletion derivatives of kalirin-7 were generated by cloning PCR-amplified sequences via *XbaI* and *NotI* in frame with an additional 5′-FLAG tag (*SpeI*/*NotI*) into pcDNA3.1 (−) (Invitrogen). All synphilin-1 constructs were obtained in the same way but with a 5′-V5 tag. Kalirin-7 constructs encompass aa 166–1663, 166–1293, 642–1663, 166–647 and 413–889, respectively. Synphilin-1 constructs encompass aa 1–348, 318–586 and 556–919. For fluorescent labeling, kalirin-7 and synphilin-1 were subcloned into pEGFP-N1 and pHcRed1-DR (Clontech), respectively. A FLAG-tagged GEF dead mutant of kalirin-7 (Kal7 dGEF) was generated with the GENEART® Site-Directed Mutagenesis System (Invitrogen) by replacing an *EcoRI*-*NotI* fragment of FLAG-kalirin-7 with an *EcoRI*-*NotI* fragment containing the mutation AATGATGCAGCG. All PCR products were verified by sequencing. The GFP-tagged constructs RhoG F37A and Rac1 T17N were gifts from Dr. Philippe Fort (Centre de Recherches en Biochimie Macromoléculaire, Montpellier, France) and Rheb D60K was from Dr. Kun-Liang Guan (Department of Pharmacology and Moores Cancer Center, University of California San Diego). FLAG-tagged HDAC6 WT and H216A/H611A double mutant constructs were kindly provided by Dr. Pang Yao (Department of Pharmacology and Cancer Biology, Duke University).

### Cell Culture, Transfection and Treatment

HEK293 cells (human embryonic kidney cell line) and HN10 cells (mouse hippocampal cell line) [47] were maintained in Dulbecco’s modified Eagle medium (DMEM) and DMEM-Glutamax (Gibco) respectively, supplemented with 10% (v/v) fetal bovine serum (FBS) and 1% penicillin/streptomycin (Gibco) at 37°C in an atmosphere of 5% CO_2._ Cells were transiently transfected using LipofectamineTM 2000 (Invitrogen) or Effectene (QIAGEN), according to the manufacturer’s instructions.

### Immunoprecipitation and Immunoblotting

For immunoprecipitation experiments, cells were lysed in RIPA buffer (150 mM NaCl, 50 mM Tris (pH 7.4), 1 mM EDTA, 0.1%SDS, 0.5% sodium deoxycholate and 1% Nonidet P-40) containing Complete protease inhibitor cocktail (Roche Molecular Biochemicals) for 30 min on ice and then centrifuged for 15 min at 13,000 rpm. Protein concentrations were determined with a Bradford protein assay (Bio-Rad) according to the manufacturer’s instructions. For immunoprecipitations, a total of 500 µg protein lysate was incubated with 30 µl of protein A/G agarose beads (1∶1 slurry, Santa Cruz) anti-FLAG Agarose Affinity Gel antibody or anti-V5 Agarose Affinity Gel antibody (1∶1 slurry, sigma), overnight at 4°C. After three washing steps with phosphate buffered saline (PBS), 15 µl of 5× Laemmli buffer were added and samples were incubated at 95°C for 5 min. Proteins were separated on 7.5% SDS-polyacrylamide gels and transferred onto polyvinylidene difluoride (PVDF) membrane (Millipore). Membranes were blocked in 5% skim milk in TBST and incubated with primary antibodies overnight at 4°C, followed by an incubation with HRP-conjugated secondary antibodies (1∶3000) for 1 h at RT. Protein signals were visualized using chemiluminescence reagents (ECL, Amersham) on Hyperfilm ECL (GE Healthcare). For densitometric analysis ImageJ software version 1.6.0 (National Institutes of Health) was used.

### Fluorescence Microscopy, Immunostaining and Quantification

Cells were cultured on glass coverslips coated with poly-L-lysine (Sigma), fixed with 4% paraformaldehyde (pH 7.4) in PBS at RT or ice cold methanol (γ-tubulin staining) at 4°C for 15 min. After three washing steps with PBS, cells were permeabilized and blocked with 0.05% Triton-X 100/10% normal donkey serum (NDS) at RT for 1 h. Subsequently, the cells were incubated with various primary antibodies overnight at 4°C. Next day, cells were washed with PBS and labeled with secondary antibodies conjugated with Cy2 (Jackson ImmunoResearch Laboratories) or anti-rabbit Alexa Fluor-647 (Molecular Probes) diluted 1∶400 in PBS (pH 7.4) with 5% NDS at 37°C for 1 h. DAPI (4′,6′-diamidino-2-phenylindole) or YO-PRO-1 (Molecular Probes) were used as counter staining. Samples were mounted with Mowiol/DABCO (1,4-diazabicyclo[2.2.2]octane, Sigma) for imaging. Confocal images were taken with an AxioImager microscope equipped with an ApoTome Imaging System (Carl Zeiss). Images were pseudocolored, merged and processed with AxioVision 4.8 software (Carl Zeiss) and ImageJ software. The level of acetylated tubulin was determined with the Image J software using the Analyze/Measure command. Using the Freehand Line tool the whole territory of each cell was outlined and the integrated density (sum of the gray values of the pixels in the selection) was measured after background subtraction.

### Live Cell Imaging

Cells were grown in Lab-Tek®II chambered coverglasses (#155382, Nalge Nunc International) and transfected with EGFP-tagged kalirin-7 and/or HcRed-tagged synphilin-1. Time-lapse imaging was performed at defined positions every 2–3 h for 72 h post-transfection using a Cell Observer**®** system (Zeiss, Germany) equipped with an Axio Observer.Z1 with 63× oil immersion objective, an ApoTome Imaging System, an AxioCamMRm (all Zeiss, Germany) and an incubation chamber (37°C and 5% CO_2_). For each time-point average intensity projections of z-stacks of 6–8 optical slides with a distance of 1.5–2.0 µm between individual z-planes were aquired. This allows the detection and follow-up of aggregates throughout the entire height of an average HEK293 cell (which is about 13 µm). The intensity of excitation was constant during the experiment. Image series were saved uncompressed and analyzed with Axio Vision 4.8 software (Zeiss, Germany). For quantification, cells were monitored at 45 h, 57 h, and 67 h post-transfection and the total number of steady state synphilin-1 aggregates was counted (n >35 cells per group). The percentage of steady state aggregates was normalized to the total number of steady state aggregates in synphilin-1 expressing cells at 45 hrs post transfection.

### Agarose Gel Electrophoresis for Resolving Aggregates (AGERA) Analysis

The AGERA protocol was modified from Weiss et al. 2008. Briefly, cells were lysed in HEPES buffer (pH 7.4) for 30 min on ice and then centrifuged at 4°C for 15 min at 13,000 rpm. The protein concentrations were measured by a Bradford assay. 2× nonreducing Laemmli sample buffer (150 mmol/L Tris–HCl pH 6.8, 33% glycerol, 1.2% SDS and bromophenol blue) was added and samples were incubated at 95°C for 5 min before being run on 2% agarose gels in SDS-PAGE running buffer (192 mmol/L glycine, 25 mmol/L Trisbase and 0.1% SDS) at 100 V until the bromophenol blue running front reached the bottom of the gel. Proteins were transferred onto PVDF membranes for 1 h at 350 mV with the Semi dry blotter PEGASUS system (Luebeck, Germany). Subsequent steps were as described above. For densitometric analysis ImageJ software version 1.6.0 (National Institutes of Health) was used and the signal was normalized to the mean expression level of controls at 24 hrs post-transfection.

### Statistical Analysis

Statistical significance (p<0.05) was assessed with paired, two-sided Student’s *t* tests. Error bars indicate the standard error of the mean (SEM). Asterisks indicate significance, ^*^ *P*≤0.05, ^**^ *P*≤0.005, ^***^ *P*≤0.001.

## Results

### Kalirin-7 Interacts with Synphilin-1 in vitro and in vivo

To explore novel binding partners of synphilin-1, we used human synphilin-1 as bait to screen a human library in the yeast two-hybrid system. Among the identified interactors were partial sequences of kalirin-7/HAPIP (aa 717–1664) and periphilin-1. The interaction of synphilin-1 with periphilin-1 was described previously [48].

**Figure 1:**
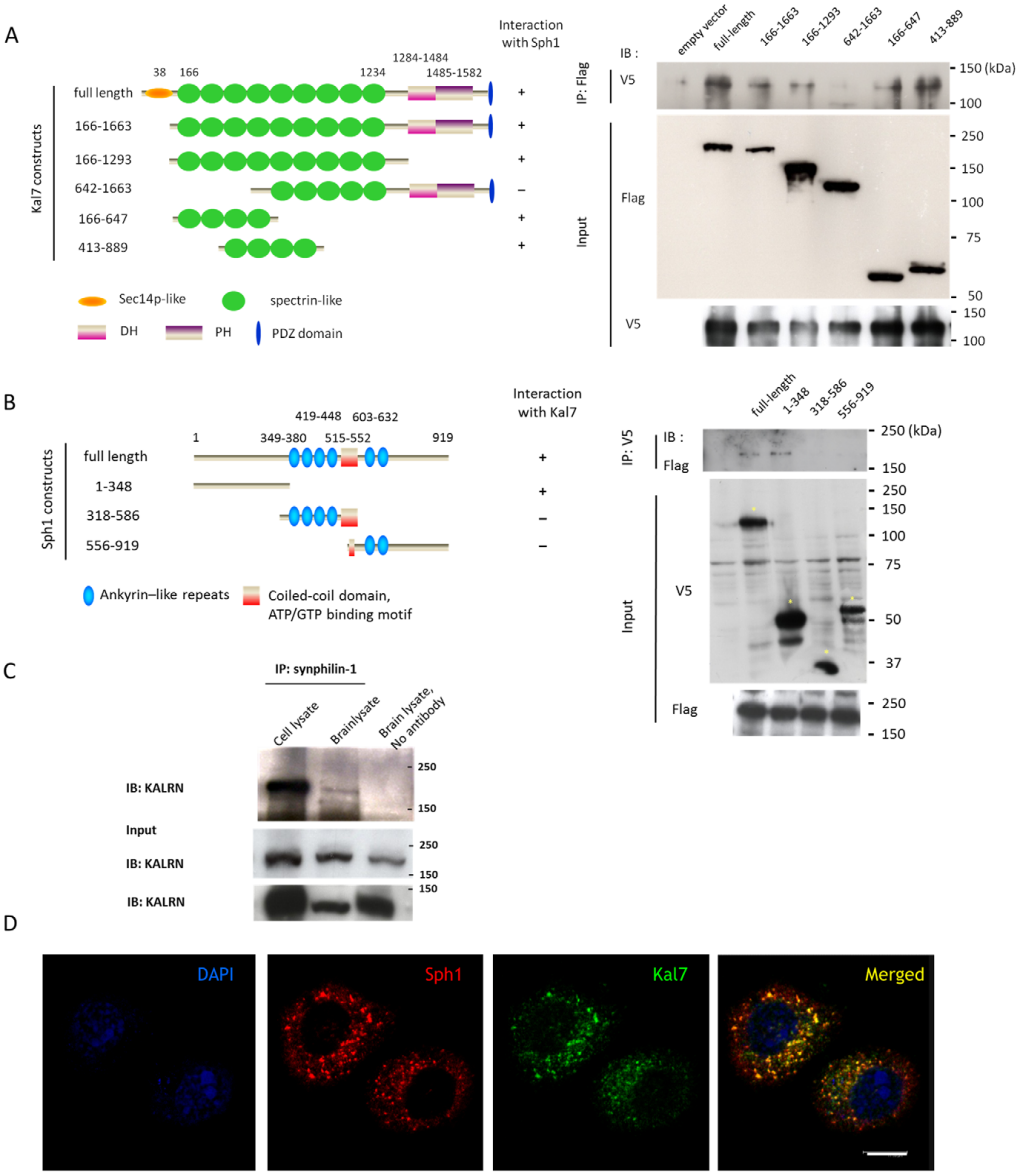
Kalirin-7 interacts with synphilin-1 *in vitro* and *in vivo*. (A) Mapping of the interacting domain in the kalirin-7 protein. FLAG-kalirin-7 constructs as shown in the diagram were co-transfected with V5-synphilin-1 in HEK293 cells. 24 h after transfection, cells were subjected to immunoprecipitation with anti-FLAG agarose beads and subsequently kalirin-7 and synphilin-1 immunoreactivities were monitored applying anti-FLAG- or anti-V5 antibodies, respectively. IP indicates antibodies used for pulling down target proteins. IB indicates antibodies used for detection in western blot. The figure shows that kalirin-7 co-immunopreciptates with synphilin-1 and that spectrin repeats III and IV of the kalirin-7 protein are crucial for the interaction. Quantification of kalirin-7 fragment expression is shown in Figure S3. (B) Mapping of the binding region in synphilin-1. The indicated V5-synphilin-1 constructs were co-transfected with FLAG-kalirin-7. Synphilin-1 fragments were precipitated with anti-V5 antibodies. The precipitates were then probed with anti-FLAG antibodies to detect co-precipitated kalirin-7. The deletion mapping revealed that amino acids 1–348 of the synphilin-1 protein are crucial for the binding of kalirin-7. The asterisks indicate specific input signals of synphilin-1 fragments. For quantification of synphilin-1 fragment expression please refer to Figure S3. (C) Endogenous synphilin-1 interacts with kalirin-7. Synphilin-1 was precipitated from whole-brain tissues (500 µg) of a wild type mouse with an anti-synphilin-1 antibody (Sigma). The precipitates were probed with a kalirin-7-specific antibody (KALRN from Abcam). Cell lysate of HEK293 cells overexpressed with FLAG-kalirin-7 and V5-synphilin-1 served as positive control. As a negative control brain lysate was subjected to immunoprecipitation without antibody. (D) Overlapping localization of kalirin-7 and synphilin-1 in cell culture. HEK293 cells were transiently transfected with both constructs for 6 h and stained with anti-FLAG and anti-V5 antibodies. The counterstaining was done with YoPro dye. The confocal sections demonstrate that both proteins display a punctate staining in the cytoplasm. Sph1, synphilin-1; Kal7, kalirin-7. *Scale bar*, 10 µm.

To confirm the interaction and to define the critical interacting domain of kalirin-7, co-immunoprecipitation experiments were performed using FLAG-tagged kalirin-7 or various deletion mutants and V5-tagged full length synphilin-1. The results showed that synphilin-1 co-precipitated with full-length kalirin-7 and that spectrin domains III and IV are necessary for the interaction with synphilin-1 (Fig. 1A and Figure S3). The kalirin-binding domain of synphilin-1 was determined accordingly by co-immunoprecipitation with different V5-synphilin-1 mutants and revealed the N-terminus of synphilin-1 (aa 1–348) as being crucial for the interaction (Fig. 1B and Figure S3). Taking into account the findings from the Y2H experiment that applied a synphilin-1 fusion construct encoding aa 177–348 and 557–920 as bait protein, the interacting domain of the synphilin-1 protein can be further narrowed down to amino acids 177–348.

To validate the interaction *in vivo* we immunoprecipitated synphilin-1 from mouse brain tissue and probed the immunoprecipitates with the KALRN antibody which recognizes isoform 2 of the kalirin protein (190 kDa), corresponding to the major adult isoform in rat brain, kalirin-7. The observed co-precipitation of kalirin-7 confirmed that endogenous synphilin-1 and kalirin-7 interact also in an *in vivo* situation (Fig. 1C).

We then investigated whether kalirin-7 co-localizes with synphilin-1 in cells when both proteins are transiently overexpressed. Laser scanning microscopy revealed that both proteins localize to the cytoplasm and show a similar cellular distribution (Fig. 1D). The results were confirmed in HEK293 cells (Fig. 1D) and neuronal HN10 cells (data not shown).

### Kalirin-7 Promotes the Recruitment of Synphilin-1 Inclusions into Aggresomes

In order to evaluate the functional relevance of the newly discovered interaction, we first focused on the ability of synphilin-1 to promote inclusion formation [9], [37], [38]. Synphilin-1 containing inclusions are often characterized as aggresomes, whose formation is dependent on retrograde dynein-dependent trafficking along microtubules. This intracellular traffic can be regulated by Rho-GTPases [45] and importantly, kalirin proteins are neuronal Rho-GDP/GTP exchange factors (GEF) and may thus be involved in this process.

**Figure 2:**
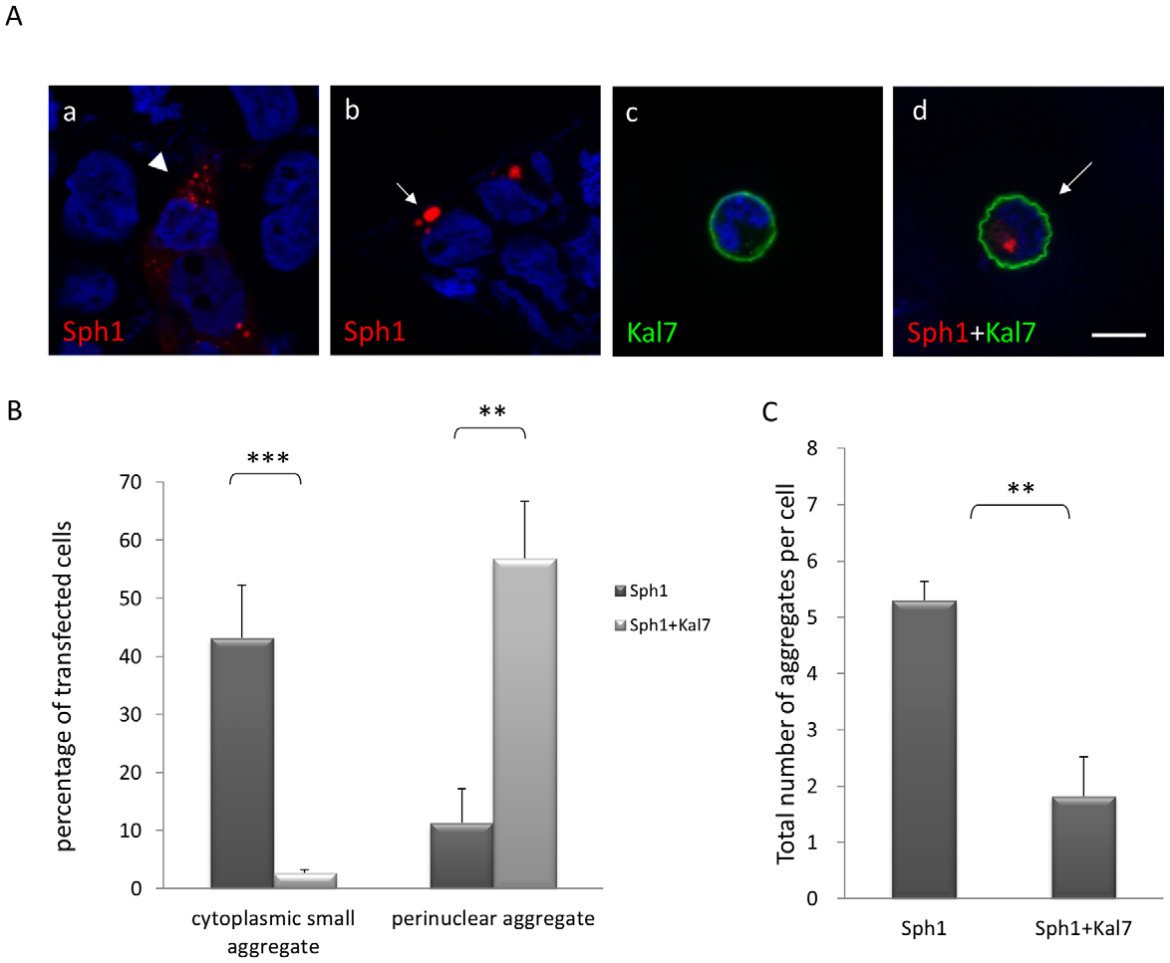
Kalirin-7 alters synphilin-1-induced inclusion formation. (A) When HEK293 cells were transfected with HcRed-synphilin-1 alone (a,b), FLAG-kalirin-7 (c) or both expression constructs (d) for 48 h, two types of inclusions were observed: small cytoplasmic aggregates (arrowhead; a) and perinuclear aggregates (arrow; b, d). *Blue*, DAPI. *Scale bar*, 10 µm. (B) Quantitative analysis of the experiment described in (A). HcRed-synphilin-1 was expressed without or with FLAG-kalirin-7 for 48 h. Cells were fixed and immunostained with anti-FLAG antibodies. Cells with cytoplasmic small aggregates, perinuclear aggregates or soluble synphilin-1 were counted. Results represent the average of three independent experiments. (C) Total numbers of aggregates per cell (cytoplasmic and perinuclear) were counted applying ApoTome confocal fluorescent microscopy. Over 100 cells were counted for each condition. The asterisks indicate statistical significance (**P≤0.005; ***P≤0.001). *Error bars*, S.E.

To investigate a potential effect of kalirin-7 on synphilin-1-induced inclusions formation, synphilin-1 tagged with HcRed was transiently overexpressed in HEK293 cells. 48 h after transfection, 43.2% of transfected cells formed cytoplasmic small aggregates, whereas 1.3% showed perinuclear aggregates (Fig. 2A and 2B; for details on aggregate classification, please refer to Figure S4). Importantly, the co-expression of FLAG-kalirin-7 resulted in a dramatic increase in the percentage of perinuclear synphilin-1 positive aggregates. 56.9% of double transfected cells formed perinuclear aggregates, but only 2.7% of transfected cells showed cytoplasmic small aggregates (Fig. 2B). Of note, HcRed alone expressed from the same vector showed a diffuse pattern and also kalirin-7 overexpression alone did not cause the formation of aggresomes up to 48 hours post transfection (Figure S2). Moreover, the average number of inclusions per cell decreased from 5.3 to 1.8 (Fig. 2C). The same results were obtained in the HN-10 cell line, a mouse hippocampal cell line (Figure S1). These data clearly demonstrate that the co-expression of kalirin-7 and synphilin-1 causes a dramatic relocation of synphilin-1 cytoplasmic small inclusions to a single prominent, perinuclear inclusion. Notably, in Figure 1D and Figure 2A we saw a dramatically different morphology of kalirin-7 at 6 h (Fig. 1D) and 48 h post-transfection (Fig. 2A and Figure S4). To further explore these differences, we monitored the distribution of kalirin-7 at various time points. 6 h after transfection kalirin-7 showed a punctate staining in the cytoplasm in line with its known association with internal membranous organelles such as the ER and the Golgi apparatus [49], while with increasing time Kal7 forms a continuous layer under a uniformly curved membrane inducing the formation of adherent compact, round cells after 24 hours (Figure S2). These observations are in agreement with a previous study [50], where Martin and colleagues could further show that the Sec14/spectrin repeat region of kalirin-7 contributes to the structural integrity of the compact, round cells.

**Figure 3:**
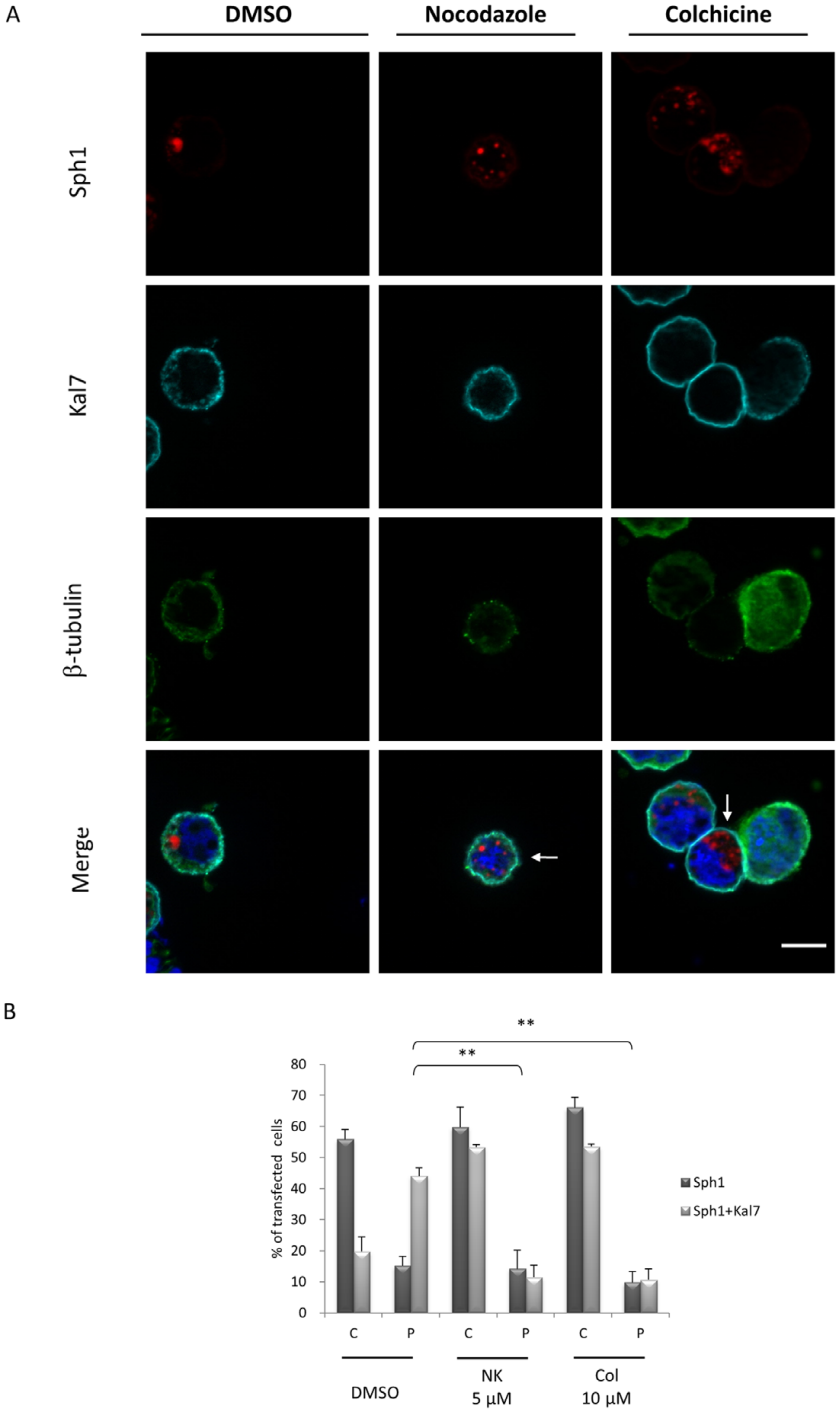
Kalirin-7 mediates perinuclear synphilin-1 inclusion formation in a microtubule-dependent manner. (A) HEK293 cells were cotransfected with HcRed-synphilin-1 and FLAG-kalirin-7. After 36 h cells were incubated with DMSO, 5 µM nocodazole or 10 µM colchicine for 12 h before being subjected to immunofluorescence with anti-FLAG and anti- β-tubulin antibodies. Cells expressing HcRed-synphilin-1 alone served as controls (arrowhead). In cells treated with nocodazole or colchicine, more cytoplasmic small aggregates (arrows) were formed. (B) Quantification (n >250 cells per group) shows that nocodazole and colchicine inhibited the kalirin-7-mediated formation of synphilin-1-containing perinuclear inclusions. P, perinuclear aggregates; C, cytoplasmic small aggregates. The asterisks indicate statistical significance (^**^ *P*≤0.005). *Error bars*, S.E.

The morphology and localization of the perinuclear inclusions appeared similar to that of aggresomes [44], [51]. It has been reported that aggresomes are formed via retrograde transport of aggregated proteins on microtubules [51]. Therefore, we first assessed the effects of microtubule-depolymerizing drugs on kalirin-7-mediated synphilin-1 perinuclear inclusion formation in HEK293 and HN-10 cells. When microtubules were disassembled by microtubule poisons nocodazole and colchicine the aggregation pattern of HcRed-synphilin-1 switched from perinuclear aggregates into small cytoplasmic aggregates (Fig. 3A). Disruption of microtubules led to a decrease in the percentage of perinuclear inclusions from 44.1% to 11.4% (nocodazole) and 10.6% (colchicine), respectively (Fig. 3B).

**Figure 4:**
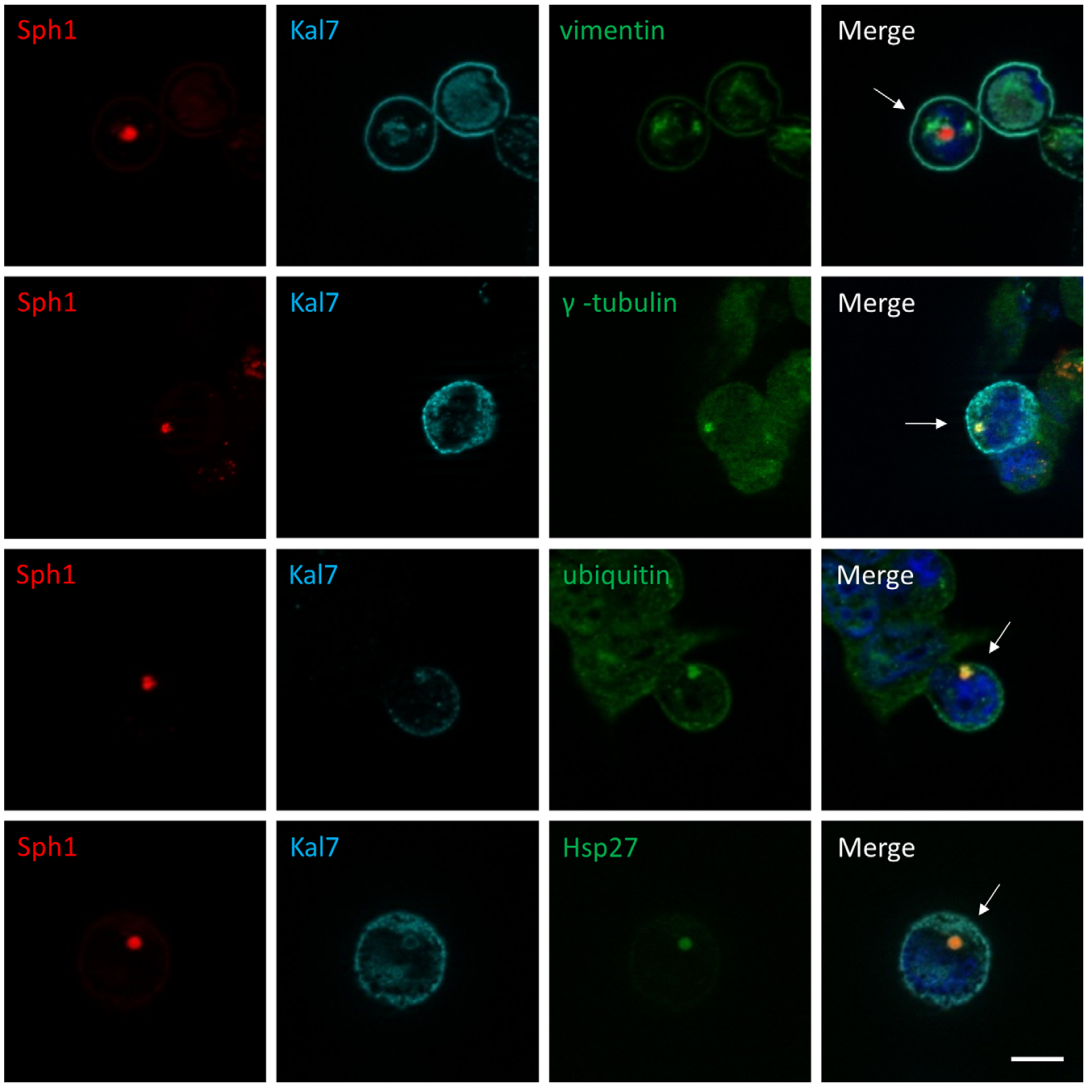
Characterization of synphilin-1-containing aggregates as aggresomes. HEK293 cells coexpressing HcRed-synphilin-1 and FLAG-kalirin-7 were fixed 48 h post-transfection and subsequently stained with the indicated antibodies. Arrows indicate the colocalization between synphilin-1 inclusions and γ-tubulin, ubiquitin and Hsp27 while the intermediate filament protein vimentin forms a cage surrounding a pericentriolar core of aggregates. Merged images are shown to the right. *Blue*, DAPI. *Scale bar*, 10 µm.

To confirm that these perinuclear inclusions were indeed aggresomes, cells were stained with antibodies against γ-tubulin, a centrosome component that is typically found in aggresomes. The immunostainings confirmed a co-localization of synphilin-1-containing perinuclear aggregates with the centrosome (Fig. 4). Other widely used markers for aggresomes, including vimentin, ubiquitin and Hsp27, were also used to characterize these inclusions. We found that the perinuclear aggregates of double transfected cells were positive for γ-tubulin, ubiquitin and Hsp27 and were surrounded by a compact cage of the intermediate filament protein vimentin (arrows in Fig. 4) and therefore fulfill all criteria of aggresomes.

### Kalirin-7 Renders Synphilin-1 Inclusions Susceptible to Degradation

**Figure 5:**
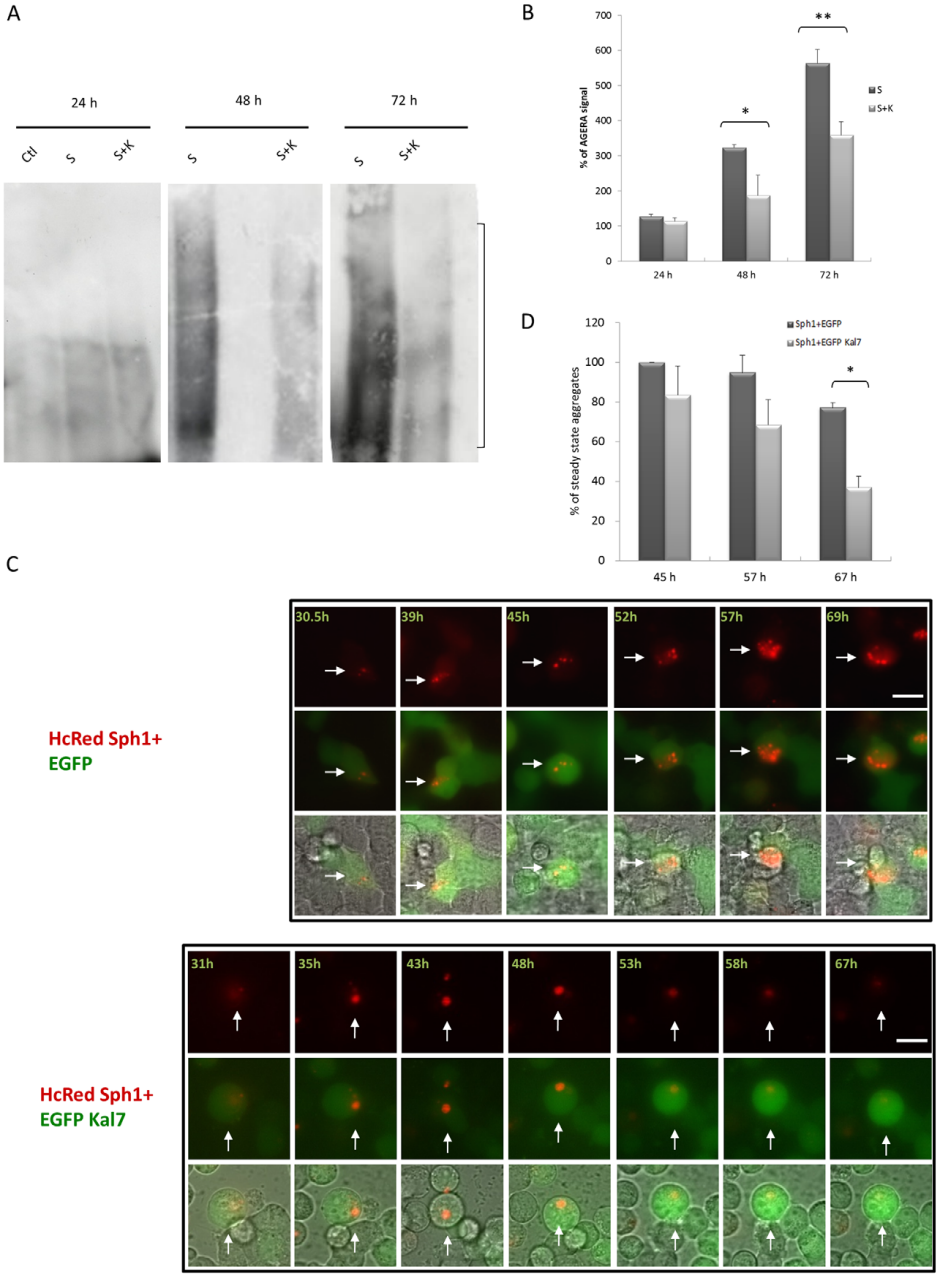
Kalirin-7 decreases synphilin-1-induced aggregates in biochemical and live cell analysis. (A) HEK293 cells were transfected with HcRed-synphilin-1 alone or cotransfected with FLAG-kalirin-7. HcRed empty vector served as control. Cells were lysed 24, 48 or 72 h after transfection, fractionated by AGERA on 2% agarose gels and analyzed by western blotting with an antibody recognizing synphilin-1 aggregates. S, HcRed-synphilin-1; K, FLAG-kalirin-7; C, control (HcRed empty vector). Indicated by a bracket on the right is the the major area of aggregate signal which was used for quantification. (B) Quantification of AGERA blots of 3 independent experiments for each time point and condition relative to the mean expression level of controls at 24 hrs post-transfection confirmed an increase of aggregates over time and a reduced number of aggregates in cells doubly transfected with kalirin-7 and synphilin-1 compared to cells transfected with synphilin-1 alone at 48 and 72 hours. (C) Long-term time-lapse imaging. HEK293 cells were transfected with HcRed-synphilin-1 and empty EGFP vector (upper chart) or EGFP-kalirin-7 (lower chart) and observed by live cell imaging fluorescent microscopy (Cell Observer, equipped with an Axio Observer.Z1 and an ApoTome Imaging System Zeiss, Germany) at 37°C. Depicted are average intensity projections of 6–8 ApoTome optical slides encompassing the entire height of the cells. Time-points indicate hours post-transfection. Images were merged from red, green and phase contrast channels. Arrows indicate the cell traced over the experimental time. *Scale bar*, 10 µm. (D) Quantification (n >35 cells per group) of the time-lapse imaging shows that aggregate numbers are reduced when FLAG-kalirin-7 is coexpressed. Light gray bars: Sph alone; dark gray bars: Sph and Kal7 coexpression. Results represent the average of three independent experiments. The asterisks indicate statistical significance (*P≤0.05). *Error bars*, S.E.

To quantify synphilin-1 inclusions biochemically, both for HcRed-synphilin-1 expression alone and for co-expression with FLAG-kalirin-7, a newly described method, Agarose gel electrophoresis for resolving aggregates (AGERA), was used [52], [53]. It is a sensitive biochemical method used to reliably quantify aggregate load and size, as demonstrated for the huntingtin protein *in vitro* and *in vivo* [54], [55]. The analyses showed that the amount of synphilin-1 aggregates increased in a time-dependent manner (Fig. 5A). As a control, the soluble forms of transfected proteins were visualised by Western blotting and comparable levels were observed at all time points (data not shown). Notably, in accordance with the results from the immunofluorescence stainings, co-expression of FLAG-kalirin-7 resulted in less aggregates than for HcRed-synphilin-1 expression alone at 48 h after transfection. The same result was obtained 72 h post-transfection (Fig. 5A). Quantification of the AGERA blots confirmed these observations (Fig. 5B).

Next, we monitored the effect of kalirin-7 on the temporal and spatial changes of synphilin-1 aggregates in living cells. For this purpose we performed time-lapse imaging, which has been used to investigate fundamental cellular processes and the formation of huntingtin aggregates in a cell model of HD [56]. Long-term monitoring revealed that cytoplasmic small aggregates were stable whereas aggresomes disappeared at the end of observation (Fig. 5C). The total number of steady state synphilin-1 aggregates was significantly reduced at 67 h post-transfection when kalirin-7 was co-overexpressed (Fig. 5D). These results indicate that kalirin-7 increases the susceptibility of synphilin-1 inclusions to be degraded.

### Recruitment of Synphilin-1 Aggregates into Aggresomes by Kalirin-7 is HDAC6 Dependent

To test if the observed enhancement of aggresome formation by kalirin-7 is dependent on its Rho-GEF activity as outlined above, we tested whether various dominant negative Rho-like small GTPases mutants, such as RhoG F37A, Rac1 T17N and Rheb D60K could inhibit the kalirin-7-mediated recruitment of synphilin-1 aggregates into aggresomes. However, none of the tested Rho-like small GTPase mutants, nor a GEF inactive mutant construct (Kal7 dGEF) led to significant changes in the percentage of cytoplasmic and perinuclear aggregates (Table 1), indicating that the enhanced formation of aggresomes does not depend on the GTP-GDP exchange activity of kalirin-7.

**Table 1:** Percent of cells with cytoplamic small aggregates or perinuclear aggregates in HEK293 cells transfected with different small GTPase competitive constructs.

Constructs	% of cytoplasmic small aggregates	% of perinuclear aggregates
Sph1	43.2±9.05	11.3±5.86
Sph1+Kal7	2.0±0.49	56.9±9.70
Sph1+Kal7+RhoG F37A*	6.31±8.26	44.17±7.65
Sph1+Kal7+Rac1 T17N*	8.71±3.99	61.36±3.21
Sph1+Kal7+Rheb D60K*	16.01±4.26	42.47±4.33
Sph1+Kal7 dGEF**	7.51±5.34	59.77±6.30

Numbers in the table represent the mean ± standard deviation.

* Cells were co-transfected with HcRed-synphilin-1, FLAG-kalirin-7 and RhoG F37A/Rac1 T17N/Rheb D60K (dominant negative RhoG/Rac1/Rheb constructs).

** Cells were co-transfected with HcRed-synphilin-1 and an inactivated GEF domain of kalirin-7.

**Figure 6:**
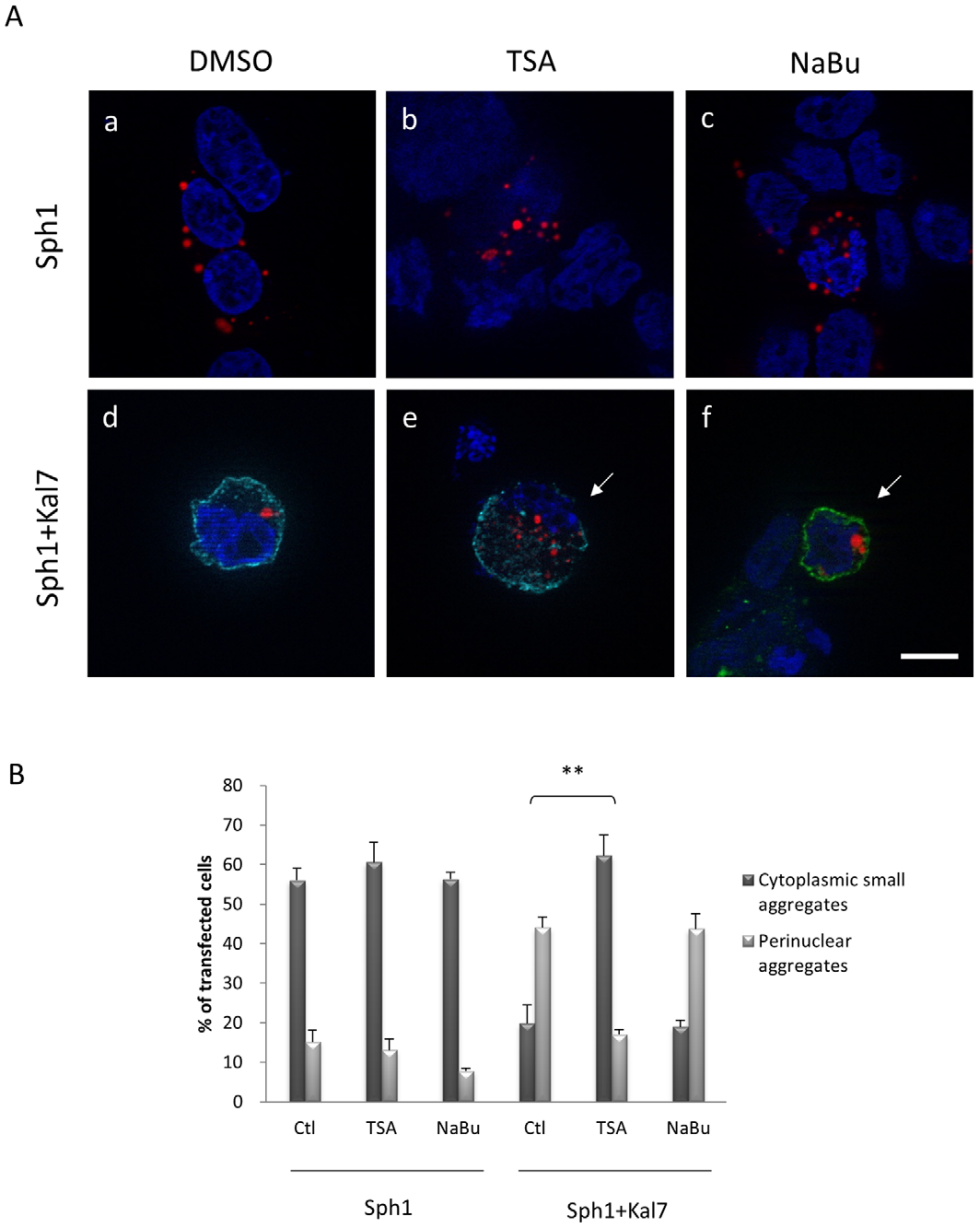
Kalirin-7-mediated recruitment of synphilin-1 inclusions into aggresome is blocked by the HDAC inhibitor trichostatin A. (A) HEK293 cells expressing HcRed-synphilin-1 (a,b,c) or co-expressing HcRed-synphilin-1 and FLAG-kalirin-7 (d,e,f) were incubated in the presence of DMSO (a,d), 1 µM TSA (b,e) or 5 mM NaBu (c,f) for 18 h before being fixed and immunostained with anti-FLAG antibodies. The arrow indicates synphilin-1 cytoplasmic small aggregates. *Blue*, DAPI. *Scale bar*, 10 µm. (B) Quantification shows that treatment with the HDAC6 inhibitor TSA counteracts the recruitment of synphilin-1 into aggresomes mediated by kalirin-7, whereas the broad deacetylase inhibitor NaBu does not exert such an effect. The asterisks indicate statistical significance (**P≤0.005). Error bars, S.E.

**Figure 7:**
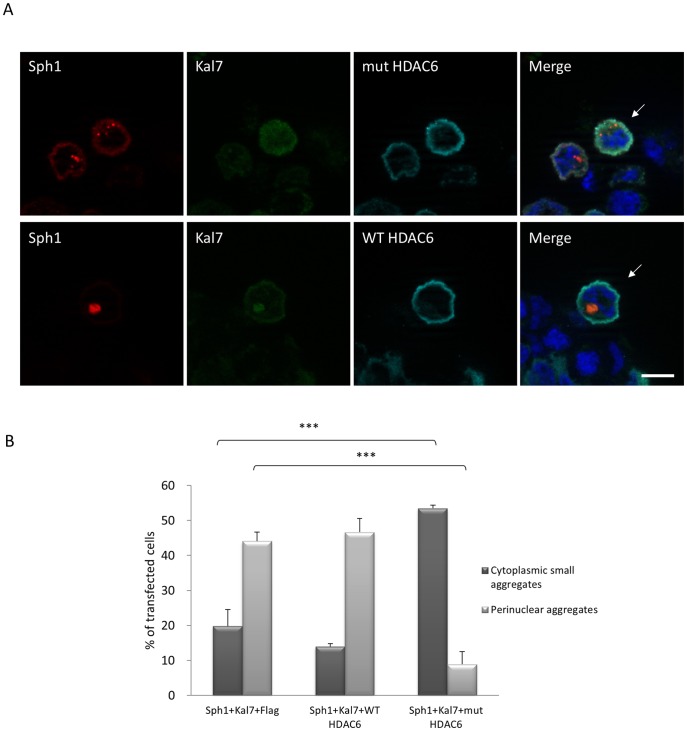
An HDAC6 deacetylase-dead mutant opposes the formation of synphilin-1 containing aggresomes mediated by kalirin-7. (A) HEK293 cells were triple-transfected with HcRed-synphilin-1, EGFP-kalirin-7 and FLAG-WT HDAC6 or FLAG-H216A/H611A mutant HDAC6. After 48 h cells were fixed and stained with anti-FLAG antibodies. Only cells co-expressing mutant HDAC6 form more cytoplasmic small aggregates (arrows). (B) Quantification shows that mutant HDAC6 inhibited the kalirin-7-mediated perinuclear synphilin-1 inclusion formation. The asterisks indicate statistical significance (^***^ *P*≤0.001). *Error bars*, S.E.

To determine the molecular basis of the observed kalirin-dependent aggresome formation, we focused on other aspects of aggresome formation and function. It is known that polyubiquitinated proteins are transported into aggresomes for degradation and/or storage. This process is mediated by histone deacetylase 6 (HDAC6) which binds both polyubiquitinated misfolded proteins and dyneins, thereby recruiting protein cargo to dynein motors for transport to aggresomes [57]. We therefore tested whether HDAC6 is involved in the kalirin-7-mediated synphilin-1 aggresome formation. To examine this, we first treated cells with trichostatin A (TSA) which inhibits three classes of HDACs, or sodium butyrate (NaBu), a broad deacetylase inhibitor which does not affect HDAC6 activity [58]. As shown in Figure 6A and 6B, treatment with TSA, but not with NaBu, resulted in an increase of small cytoplasmic aggregates when HcRed-synphilin-1 and FLAG-kalirin-7 were co-expressed. The percentage of perinuclear aggregates in double transfected cells decreased from 44.1% to 17.03% after treatment with 1 µM TSA (Fig. 6B). Thus TSA significantly inhibited the kalirin-7-mediated recruitment of synphilin-1 aggregates into aggresomes. HDAC6 contains two deacetylase domains that are both required for normal deacetylase activity [59]. To further explore whether the kalirin-7-induced aggresome response is dependent on the catalytic activity of HDAC6, WT and double mutant (H216A/H611A) HDAC6 were triple transfected with synphilin-1 and kalirin-7 to compete with the endogenous HDAC6 protein (Fig. 7A). The percentage of cytoplasmic small aggregates in the mutant HDAC6 triple transfected cells significantly increased from 19.79% to 53.49% (Fig. 7B), while HDAC6 WT did not affect the aggresome response. These data underline that kalirin-7 stimulates the recruitment of synphilin-1 into aggresomes in an HDAC6-dependent manner.

**Figure 8:**
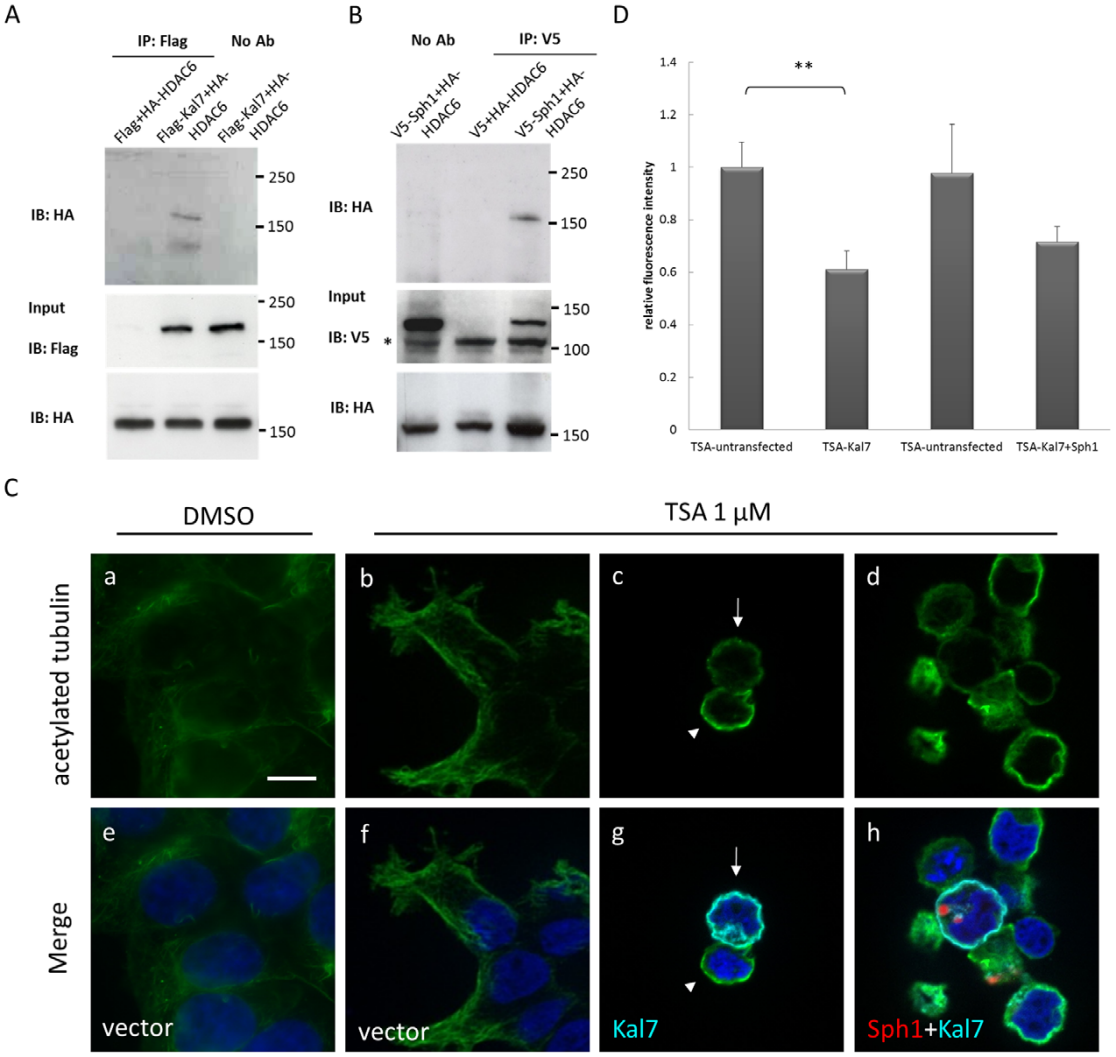
Decreased tubulin acetylation in kalirin-7 expressing cells under TSA treatment. (A, B) An interaction of FLAG-kalirin-7, V5-synphilin-1 and HA-HDAC6 was examined by co-immunoprecipitation experiments. 24 h after transfection, HEK293 cells were lysed and 500 µg of protein lysates were subjected to immunoprecipitation with anti-FLAG or anti-V5 conjugated agarose beads, respectively. The precipitates were probed with HA antibodies to detect HDAC6 and revealed an interaction of HDAC6 with both kalirin-7 and synphilin-1. 30 µg of protein lysates were visualized as input control. The asterisk indicates a non-specific band observed in all raw lysates detected with anti-V5. (C) Cells transiently overexpressing FLAG-kalirin-7 (c, g), FLAG-kalirin-7 plus HcRed-synphilin-1 (d, h), or empty vectors (a, b, e, f) were immunostained for acetylated tubulin (a-d, green) and kalirin-7 (g, h light blue) after DMSO or 1 µM TSA treatment. While TSA treatment resulted in higher acetylation levels in comparison to controls (arrowheads), the overexpression of kalirin-7 led to a significant decrease of the α-tubulin acetylation levels (arrows). Blue, DAPI. Scale bar, 10 µm. (D) Acetylated tubulin levels were quantified by the fluorescence signal of individual cells, as described in Materials and Methods. Kalirin-7 transfected cells treated with TSA were compared to untransfected cells in the same cell population. Comparably, kalirin-7/synphilin-1 doubly transfected cells treated with TSA were quantified relative to untransfected cells in the same population. The asterisks indicate statistical significance (^**^ *P*≤0.005). *Error bars*, S.E., n = 100.

Apart from binding to ubiquitinated proteins, HDAC6 catalyzes the removal of acetyl groups from α-tubulin and plays an important role in microtubule-dependent intracellular trafficking [60]. Given this involvement, we next tested if kalirin-7, synphilin-1 and HDAC6 act in a common complex to transport synphilin-1 into aggresomes. To address this hypothesis, we first tested if kalirin-7 and HDAC6 were in a protein complex by means of coimmunoprecipitation experiments. As shown in Figure 8A, kalirin-7 and HDAC6 were in a protein complex. In addition, an interaction between synphilin-1 and HDAC6 was observed (Fig. 8B).

**Figure 9:**
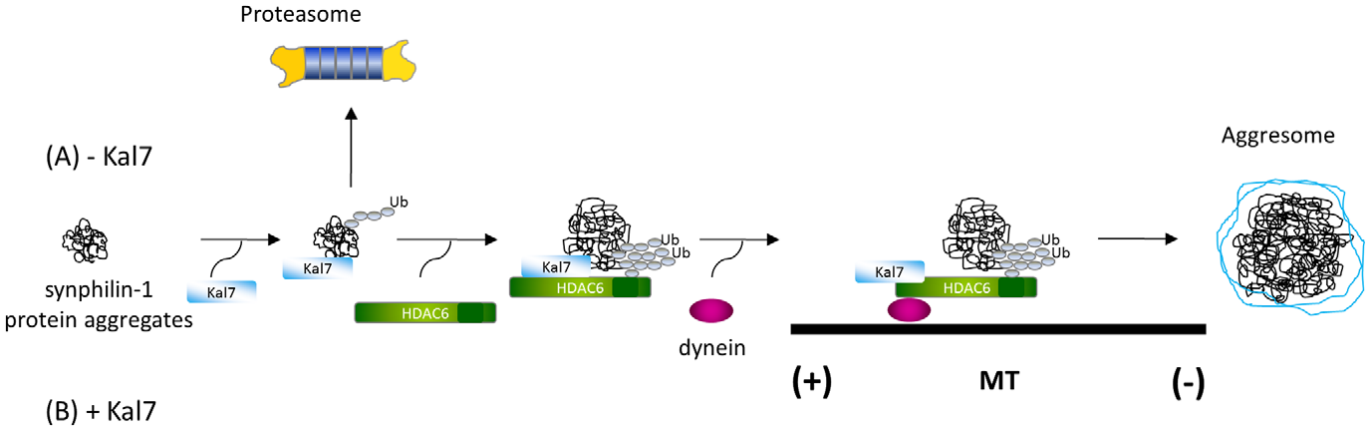
Proposed pathway of kalirin-7-mediated synphilin-1 aggresome formation. (A) Under normal conditions, misfolded synphilin-1 is mainly accumulated in cytoplasmic small aggregates. (B) When kalirin-7 is overexpressed, it facilitates the recruitment of HDAC6 and the dynein motor complex and acts on microtubule dynamics by stimulating the deacetylase activity of HDAC6, thereby increasing the transportation of synphilin-1 into aggresomes.

Next, we evaluated if kalirin-7 would affect the acetylation level of α-tubulin. The changes in tubulin acetylation were quantified by measuring the fluorescence intensity of individual cells after immunostaining for acetylated tubulin in HEK cells overexpressing empty vector, FLAG-kalirin-7 or FLAG-kalirin-7 plus HcRed-synphilin-1. While TSA treatment resulted in higher acetylation levels in comparison to controls, the presence of kalirin-7 led to a significant decrease of the acetylation level of α-tubulin compared to untransfected cells (Fig. 8C–D), indicating that kalirin-7 activates the deacetylation activity of HDAC6. This could represent the molecular basis for the kalirin-mediated effect on the microtubule-dependent recruitment of synphilin-1 inclusions into aggresomes. The proposed mechanism is shown in Figure 9.

## Discussion

In the current study, we have identified kalirin-7 as a novel interactor of synphilin-1 and were able to determine a functional implication of kalirin-7 in the aggregation behavior of synphilin-1.

Synphilin-1 is linked to the pathogenesis of Parkinson disease by its presence in Lewy bodies [61], its interaction with α-synuclein, a key protein of PD, its relation to the UPS both as a substrate and a mediator of degradation [14], [16], [62], and its involvement in synaptic vesicle trafficking [63]. We have previously identified periphilin as an interactor of synphilin-1 which displayed overlapping expression patterns in cells, animals and Lewy bodies of PD patients [48]. In this study, we describe for the first time the interaction between synphilin-1 and the brain specific Rho GEF kalirin-7. We found that kalirin-7 enhanced the recruitment of synphilin-1 inclusions into aggresomes and their subsequent degradation. The action of kalirin-7 was not related to its well known GEF activity but rather an HDAC6-mediated process.

The interacting domains of synphilin-1 and kalirin-7 were narrowed down to an N-terminal region of the synphilin-1 protein (aa 177–348) and the spectrin domains III and IV in the kalirin-7 protein (aa 413–642). The interacting domain in the synphilin-1 protein therefore differs from other synphilin-1 interactors, which bind mainly to the central part of the protein, namely α-synuclein, parkin and dorfin [12], [37], [64], or to the C-terminus of synphilin-1, including acidic phospholipids and periphilin [48], [65]. Solely the minimal binding regions of the E3 ligases SIAH-1 and SIAH-2 is also located in the N-terminus, where the interacting regions (aa 1–202 for SIAH1 and 1–227 for SIAH2) overlap with the kalirin-7 binding domain [10], [11]. A recent NMR study proposed that the central coiled-coil domain (CC) of synphilin-1 forms a dimer and facilitates the self aggregation of synphilin-1 and the inclusion formation with α-synuclein [37]. In addition, the central ankyrin-like domains (ANK) encompass an aggresome-targeting signal (ANK1) and an aggregation-promoting segment (CC-ANK2) [39]. Stereologically, with the N-terminal stretch of the synphilin-1 protein being implicated in the interaction with kalirin-7, the central synphilin-1 domains would be available for the recruitment of other protein complexes and the integration of further signaling pathways.

In the current study we demonstrated that synphilin-1 predominantly forms small cytoplasmic aggregates while perinuclear aggregates increase when kalirin-7 is co-expressed (Fig. 2B). The presence of several marker proteins identified these perinuclear inclusions as aggresomes and we were able to show that synphilin-1 containing aggresomes are readily degraded while small cytoplasmic inclusions are more stable (Fig. 5).

Some data have been published in the literature concerning the nature of synphilin-1 aggregates and their relevance. Synphilin-1 is known to form multiple cytoplasmic aggregates in naïve cells, whereas in the presence of the proteasome inhibitor MG132 aggresomes are formed which are hypothesized to be cytoprotective [39], [66], [67]. There is evidence that these synphilin-1 aggresomes are ubiquitin positive. Consistent with previous reports, we also observed a co-localization of synphilin-1 and ubiquitin in aggresomes triggered by kalirin-7 (Fig. 3). The ubiquitination of synphilin-1 has been proposed to play a role in its aggregation and aggresome formation [68] and this process was mediated by parkin, an E3 ligase which promotes a K63-linked polyubiquitination of synphilin-1 [17].

Central to our study was the question how kalirin-7 could contribute to the transport of synphilin-1 into aggresomes. We first focused on the GEF activity of kalirin-7 for Rho-GTPases, especially Rac1 and RhoG [49], [69], as a Ras family GTPase, Rheb, was recently shown to modulate aggresome formation [70] which might be a potential effector of kalirin-7 albeit their interaction has not been investigated. To determine whether the GEF activity of kalirin-7-mediated the recruitment of synphilin-1 into aggresomes, we tested dominant-negative variants of Rho-like small GTPases and a dead mutant of the kalirin-7 GEF domain to block or attenuate kalirin-7-GEF activity, respectively. Unexpectedly, our results showed that the transportation of synphilin-1 into aggresomes is GEF-independent (Table 1).

Indeed, kalirin-7 is known to also have GEF-independent functions, e.g. the induction of lamellipodia does not require GEF activity [71], and comparably its effect on aggresome formation appears to act through GEF-independent pathways or in combination with accessory factors. In search for the downstream target of kalirin-7 in terms of aggresome formation, we next focused on HDAC6. This histone deacetylase has been shown to concentrate in Lewy bodies of PD patients [57] and there is increasing evidence that HDAC6 plays an essential role in aggresome formation via its ubiquitin binding and its deacetylase activity. HDAC6 binds to ubiquitinated proteins, including CFTR [57] and huntingtin [72], through a C-terminal ubiquitin-binding zinc finger domain (ZnF-UBP) and also binds to the dynein motor complex through a segment between two catalytic domains [57] facilitating the transport of cargo proteins by the dynein motor complex towards the minus end of microtubules to the microtubule-organizing center (MTOC). Furthermore, HDAC6 has been shown to mediate the deacetylation of α-tubulin in several studies [60], [73]–[75] and the inhibition of HDAC6 has been proposed to regulate the dynamics of the microtubule network [76]. A recent *in vivo* study demonstrated that the depletion of *Hdac6* in the R6/2 mouse model resulted in an increase of tubulin acetylation, but had no effect on the onset and progression of the HD-related phenotype [77]. Here, we observed that TSA (but not NaBu) as well as a dominant negative HDAC6 mutant conferring loss of catalytic activity counteracted the kalirin-7-mediated recruitment of synphilin-1 aggregates into aggresomes (Figs. 6 and 7). Importantly, kalirin-7 decreased α-tubulin acetylation induced by TSA (Fig. 8C, 8D), further corroborating an effect of kalirin-7 on HDAC6-mediated protein transport and aggresome formation.

Compatible with a concerted action of kalirin-7 and HDAC6, we showed that kalirin-7 and synphilin-1 both interact with HDAC6 (Fig. 8A, 8B), indicating that they act in a common protein complex. Taken together, one possible explanation for the efficient transport of synphilin-1 into aggresomes is that the deacetylation of α-tubulin increases the dynamics of microtubules thereby accelerating microtubule-based transport [74], [78]. Nevertheless, the relationship between tubulin acetylation and microtubule-based transport is controversial from various studies. For instance, Dompierre and coworkers showed that tubulin acetylation has no effect on microtubule dynamics in COS7 cells [79] while Reed and coworkers found an association between tubulin acetylation and microtubule stability promoting the recruitment of kinesin and dynein motors to microtubules and cargo transport [79], [80]. These data suggest that the regulation of tubulin acetylation and microtubule-based transport may depend on the cellular conditions [81]. Besides the HDAC6 dependence of the kalirin-7-mediated aggresome formation, the kalirin-7 interactor HAP-1 may act as an additional mediator or cofactor [20] of kalirin action. In its capacity as interactor of the microtubule-dependent trafficking proteins dynactin p150 [82], [83] and kinesin light chain 2 [84] that facilitate cargo transport along microtubules, HAP-1 could also play a role in the transport of synphilin-1 aggregates into aggresomes.

### Conclusions

In conclusion, this study demonstrates for the first time an interaction between synphilin-1 and kalirin-7 that leads to the recruitment of synphilin-1 into aggresomes in a GEF-independent but HDAC6-dependent manner. This is also the first report linking kalirin-7 to microtubule dynamics. Importantly, this novel interaction and its impact on aggresome formation, a fundamental mechanism in the pathogenesis of PD, potentially link kalirin-7 for the first time to PD in an *in vitro* model. Based on our findings, further *in vivo* studies are needed to prove an implication of kalirin-7 as a pathogenesis factor to PD.

## Supporting Information

Figure S1 **Kalirin-7 alters synphilin-1 induced inclusions formation in HN-10 cells.** (A) In HN-10 cell lines, HcRed-synphilin-1 was transfected without or with Flag-kalirin-7 for 48 h, fixed, and immunostained with Flag antibody. Cells with cytoplasmic small aggregates (arrowhead), perinuclear aggregates (arrow) or soluble synphilin-1 were counted. *Blue*, DAPI. *Scale bar*, 10 µm. (B) Total numbers of aggregates per cell (cytoplasmic and perinuclear) were counted applying ApoTome confocal fluorescent microscopy. Over 100 cells were counted for each condition. Results were the average of three independent experiments. The asterisks indicate statistical significance (^**^ *P*≤0.005; ^***^ *P*≤0.001). *Error bars*, S.E.
(TIF)

Figure S2 **Time course of Kalirin-7 overexpression patterns.** FLAG-kalirin-7 was overexpressed in HEK293 cells and fixed at six different time points indicated above. The samples were stained with FLAG antibody to visualize kalirin-7 expression. Merged images and bright field images are shown to the middle and right, respectively. Kalirin-7 was diffusely distributed in the cytoplasm at 6 h post-transfection whereas the protein was observed in the periphery of the nucleus 12 h after transfection. *Green*, kalirin-7; *Blue*, DAPI. *Scale bar*, 10 µm.
(TIF)

Figure S3 **Quantification of the expression of kalirin-7 and synphilin-1 protein fragments.** (A) In order map the critical interacting domain of kalirin-7 the indicated FLAG-kalirin-7 deletion constructs were co-transfected with V5-synphilin-1 in HEK293 cells and subjected to co-immunoprecipitation as detailed in the main manuscript. The expression of each fragment was quantified. (B) V5-synphilin-1 deletion constructs were used to map the critical interacting domain in the synphilin-1 protein. Expression levels were quantified accordingly.
(TIF)

Figure S4 **Morphology of two categories of synphilin-1 aggregates.** Small cytoplasmic aggregates and perinuclear aggregates were distinguished according to their size and subcellular localization. Small cytoplasmic aggregates are of a much smaller size compared to perinuclear aggregates. Only large centrosome-localized protein aggregates were identified as perinuclear aggregates.
(TIF)
