# Supplementary figures and images for: The Guanine Nucleotide Exchange Factor Kalirin-7 Is a Novel Synphilin-1 Interacting Protein and Modifies Synphilin-1 Aggregate Transport and Formation
Source: PLoS One. 2012 Dec 20;7(12):e51999. doi: 10.1371/journal.pone.0051999 (PMC3527391; doi:10.1371/journal.pone.0051999)

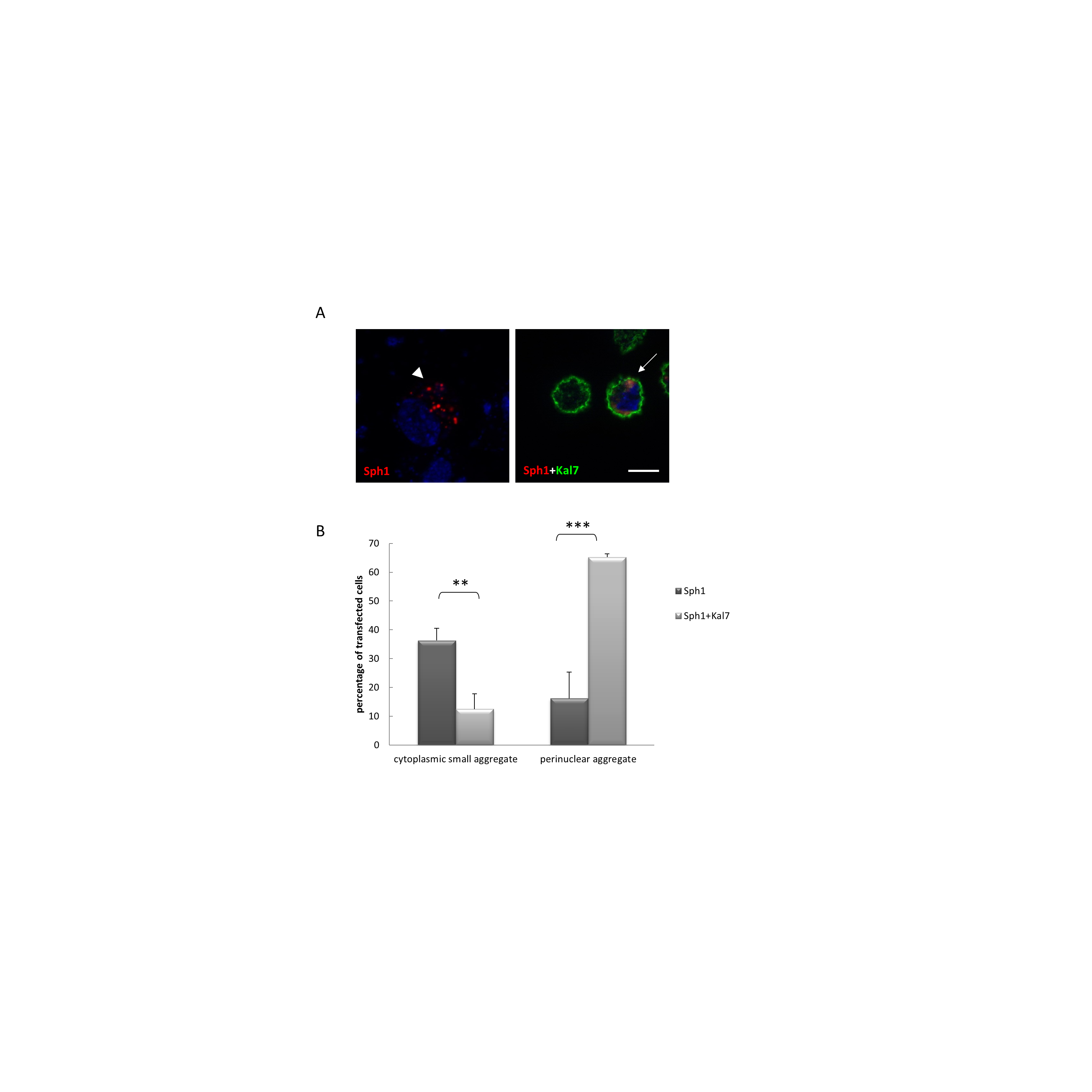

Supplement: Figure S1 — Kalirin-7 alters synphilin-1 induced inclusions formation in HN-10 cells. (A) In HN-10 cell lines, HcRed-synphilin-1 was transfected without or with Flag-kalirin-7 for 48 h, fixed, and immunostained with Flag antibody. Cells with cytoplasmic small aggregates (arrowhead), perinuclear aggregates (arrow) or soluble synphilin-1 were counted. Blue, DAPI. Scale bar, 10 µm. (B) Total numbers of aggregates per cell (cytoplasmic and perinuclear) were counted applying ApoTome confocal fluorescent microscopy. Over 100 cells were counted for each condition. Results were the average of three independent experiments. The asterisks indicate statistical significance (** P≤0.005; *** P≤0.001). Error bars, S.E. (TIF) [file pone.0051999.s001.tif]

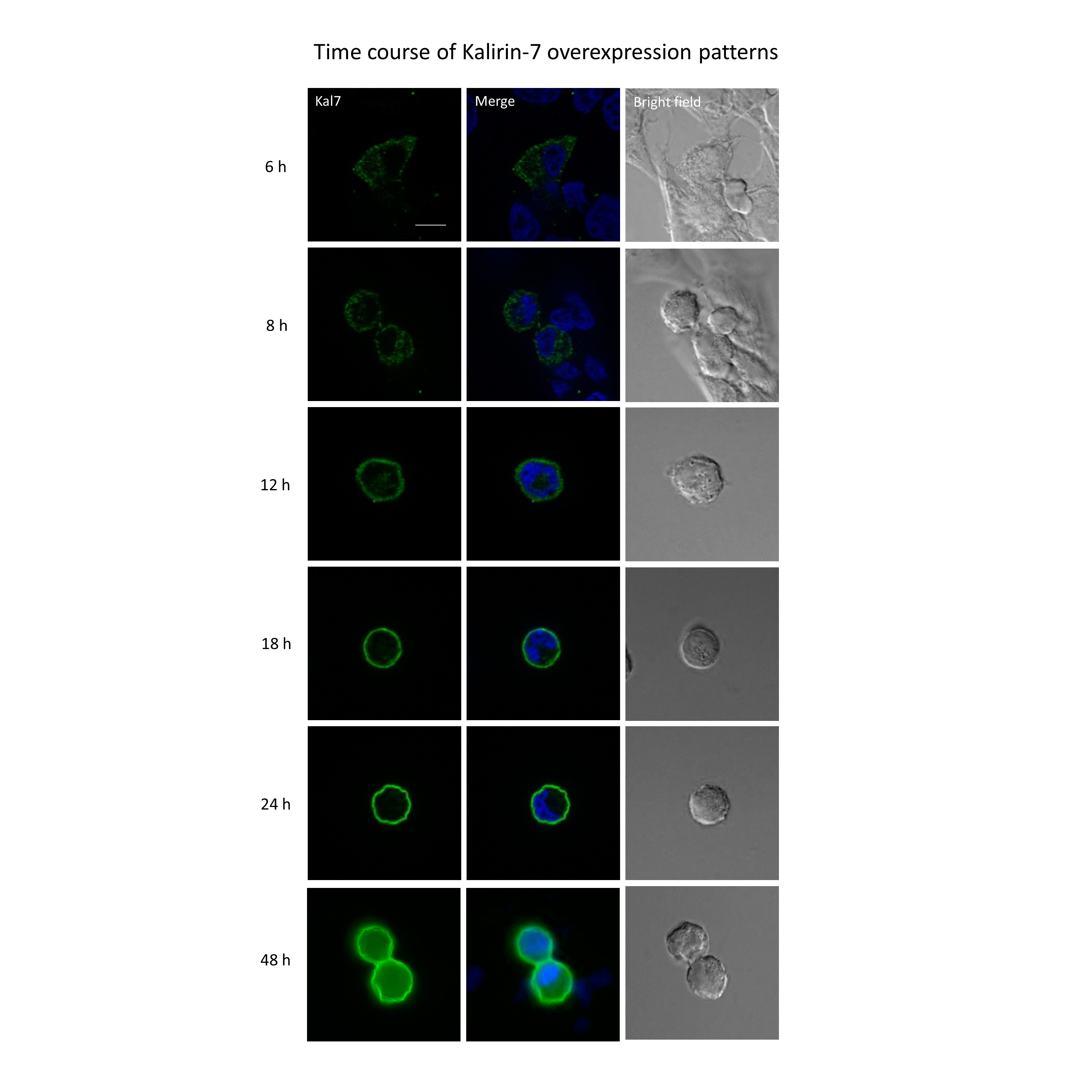

Supplement: Figure S2 — Time course of Kalirin-7 overexpression patterns. FLAG-kalirin-7 was overexpressed in HEK293 cells and fixed at six different time points indicated above. The samples were stained with FLAG antibody to visualize kalirin-7 expression. Merged images and bright field images are shown to the middle and right, respectively. Kalirin-7 was diffusely distributed in the cytoplasm at 6 h post-transfection whereas the protein was observed in the periphery of the nucleus 12 h after transfection. Green, kalirin-7; Blue, DAPI. Scale bar, 10 µm. (TIF) [file pone.0051999.s002.tif]

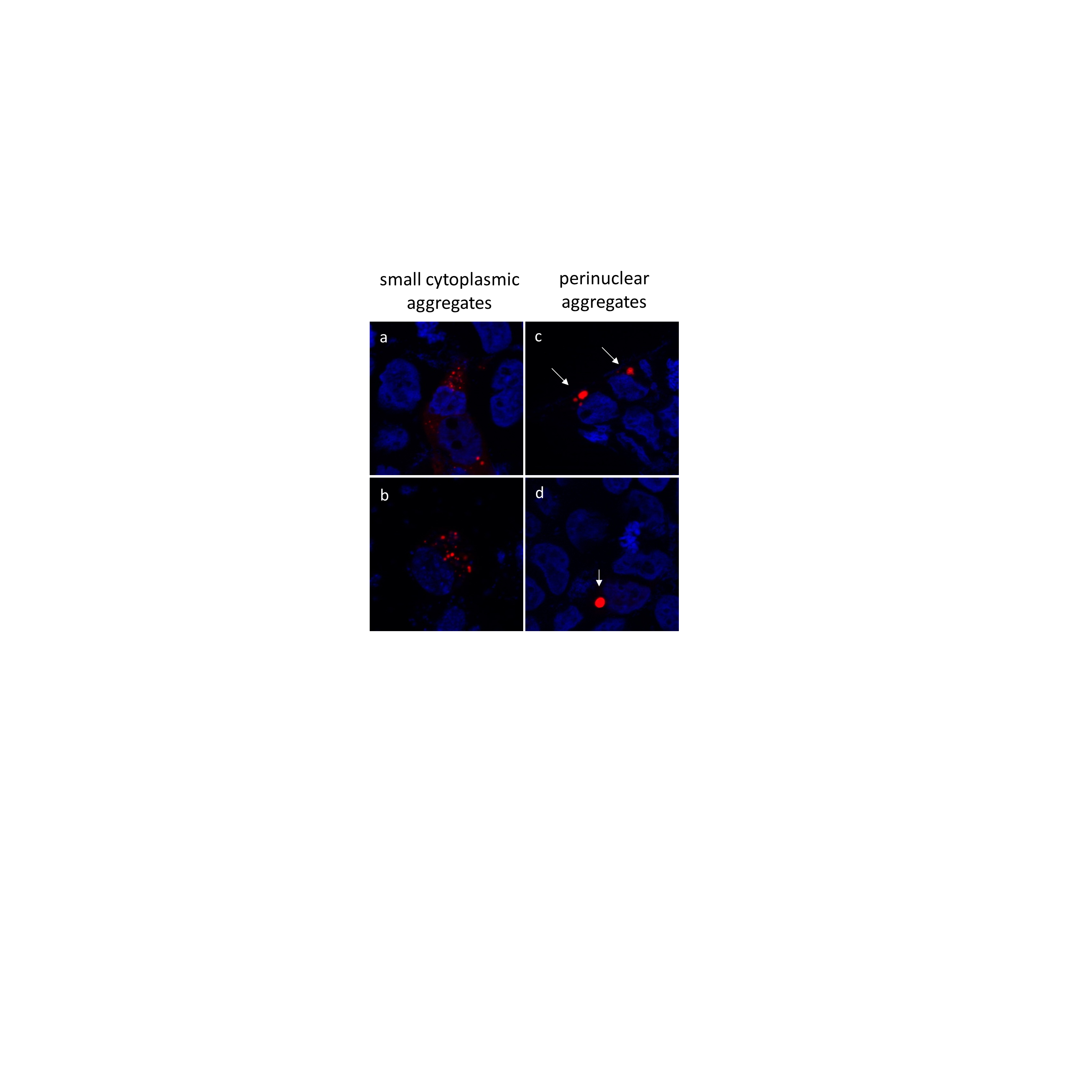

Supplement: Figure S4 — Morphology of two categories of synphilin-1 aggregates. Small cytoplasmic aggregates and perinuclear aggregates were distinguished according to their size and subcellular localization. Small cytoplasmic aggregates are of a much smaller size compared to perinuclear aggregates. Only large centrosome-localized protein aggregates were identified as perinuclear aggregates. (TIF) [file pone.0051999.s004.tif]
